# The Impact of Augmented Renal Clearance on Vancomycin Pharmacokinetics and Pharmacodynamics in Critically Ill Patients

**DOI:** 10.3390/jcm13082317

**Published:** 2024-04-17

**Authors:** Novel Solomon Tesfamariam, Asma Aboelezz, Sherif Hanafy Mahmoud

**Affiliations:** 1 Department of Pharmacy, Uppsala University, SE 751 23 Uppsala, Sweden; novelsolomon.tesfamariam.5260@student.uu.se; 2 Faculty of Pharmacy and Pharmaceutical Sciences, University of Alberta, Edmonton, AB T6G 2E1, Canada; aboelezz@ualberta.ca

**Keywords:** vancomycin, augmented renal clearance, pharmacodynamics, pharmacokinetics, creatinine clearance

## Abstract

Augmented renal clearance (ARC), defined as a creatinine clearance (CrCl) > 130 mL/min/1.73 m^2^, is observed in 30–65% of critically ill patients. When following standard dosage guidelines, patients with ARC often experience subtherapeutic vancomycin levels, resulting in treatment failure due to accelerated drug elimination. This review aims to explore ARC’s impact on vancomycin pharmacokinetics and pharmacodynamics (PK/PD) indices in ARC patients, seeking to identify an accurate dose adjustment method for this patient population. In September 2023, a comprehensive literature search was conducted on the MEDLINE and EMBASE databases to include all available studies providing information on the impact of ARC on vancomycin therapy in critically ill adults. Articles that studied the pediatric population and those with insufficient PK data were excluded. A total of 21 articles met the inclusion criteria. The findings revealed a positive correlation between CrCl and vancomycin clearance, indicating low serum concentrations. Therefore, upward dosing adjustments are necessary to improve treatment success. Younger age consistently emerged as a major contributor to ARC and vancomycin PK/PD alterations. This study summarizes the PK/PD alterations, current dosage recommendations and proposes preliminary recommendations on possible dosing approaches to decrease the risk of subtherapeutic exposure in this patient population.

## 1. Introduction

Hospital-acquired infections are common in the intensive care unit (ICU), contributing to extended hospital stays and increased patient mortality rates. Consequently, antimicrobial utilization in the ICU is 5–10 times higher than in other units [1]. Thus, ensuring early administration and achieving optimal serum concentrations of these antimicrobial agents are crucial for effective infection management. However, this task has long been challenging due to the diverse range of pathophysiological changes frequently observed in critically ill patients. These pathophysiological shifts arise from the underlying severe acute or chronic conditions as well as the responses to clinical interventions provided. They often coincide with an increase in cardiac output, resulting in increased clearance, inflammatory responses, and capillary leaks that further contribute to excess extravascular fluid [2,3]. These factors significantly impact the pharmacokinetics and pharmacodynamics (PK/PD) properties of antimicrobial drugs, making therapy outcomes unpredictable. Moreover, given that renal elimination is the main route of clearance for many commonly prescribed antimicrobials, any alteration in renal function substantially affects the pharmacokinetics of these antimicrobials, especially those with higher hydrophilicity [1,2]. The most frequently used antimicrobials in the ICU fall into this category, including beta-lactams, aminoglycosides, and vancomycin.

Pharmacokinetics (PK) refers to the process that results in the delivery of the drug to its target site. Absorption, distribution, metabolism, and elimination are fundamental aspects of understanding how the drug navigates through the body. Volume of distribution (V_d_), half-life (t_1/2_), and clearance (CL) are some of the most important parameters used to describe the active processes of pharmacokinetics. Antimicrobial pharmacodynamics (PD), on the other hand, describes the relationship between drug concentration and antimicrobial effect. In the case of antimicrobials, this is typically measured as the minimum inhibitory concentration (MIC), which is defined as the lowest concentration of an antimicrobial agent that inhibits the visible bacterial growth in a standard medium after incubation [4]. 

Several pathological conditions observed in critically ill patients, such as traumatic injuries, burns, and sepsis, exhibit hyperdynamic states and increased cardiac output, leading to augmented blood flow to major organs. Consequently, this results in elevated glomerular filtration and increased clearance of commonly used antimicrobial drugs [4]. Augmented renal clearance (ARC), typically defined as enhanced renal clearance above 130 mL/min/1.73 m^2^, is a recently described common phenomenon and represents one of the pathophysiological changes in critical care settings. It significantly impacts the optimal management of infections and, subsequently, the duration of hospitalization [5]. While our current understanding of the ARC pathophysiology remains limited, it has been associated with elevated glomerular filtration. The incidence of ARC was reported to range from 30% to 65% among ICU patients, and it increases to around 50% to 85% within specific patient populations, such as patients with sepsis or trauma [6,7]. Other common risk factors associated with ARC include young age, male sex, and the absence of comorbidities [6,8]. The impact of ARC on PK/PD indices is notably significant for antimicrobials with time-dependent activity and short half-lives. Antibiotics like vancomycin depend on the magnitude of exposure over time for their antibacterial efficacy [9]. Recent studies have revealed that patients with ARC tend to have lower concentrations of vancomycin and require higher dosages to achieve optimal exposure [10,11,12].

Vancomycin has been widely used for many years due to its effectiveness against severe Gram-positive infections caused by methicillin-resistant *Staphylococcus aureus* (MRSA) and serves as the first-line therapy for such infections in the ICU. It is a hydrophilic glycopeptide antibiotic that is eliminated renally by 80–90%, and its clearance is about 2.64 L/h [13]. It exhibits both concentration- and time-dependent activity, which is expressed as the ratio of the area under the concentration-time curve over 24 h to the MIC (AUC_24_/MIC) [14]. Monitoring the AUC is recommended along with regular monitoring of its trough concentration (C_trough_) to ensure therapeutic activity and prevent toxicity [15]. The main adverse effects of vancomycin are hypersensitivity, nephrotoxicity, and ototoxicity [9]. Because vancomycin has a narrow therapeutic range, therapeutic drug monitoring (TDM) is recommended to maximize its efficacy and minimize the risk of toxicities. However, even with TDM available, achieving the ideal vancomycin serum levels in critically ill patients with ARC remains a challenging task, and therapeutic failure occurs relatively frequently in this patient population. Therefore, adjustments to vancomycin dosing are necessary to achieve desired therapeutic outcomes, primarily relying on renal function, which is often estimated using the Cockcroft-Gault (CG) formula. However, the accuracy of the formula tends to be less reliable in ICU patients, particularly those with ARC [16,17]. Thus, there is a pressing need for clearer guidelines on vancomycin dosing in critically ill patients with ARC to mitigate the risk of subtherapeutic exposure, especially given the critical time sensitivity of these patients.

The aim of this study was to comprehensively summarize and appraise the currently available evidence regarding the dosing, safety, and efficacy of vancomycin in critically ill adult patients experiencing ARC. By doing so, we provided suggested dosing recommendations for vancomycin in patients exhibiting ARC.

## 2. Materials and Methods

### 2.1. Search Strategy

A comprehensive literature search of the databases MEDLINE and EMBASE was carried out on 26 September 2023. To capture all relevant evidence, appropriate search terms related to vancomycin therapy in critically ill patients involving ARC were used. The search included keywords such as (“Augmented renal clearance” OR “ARC” OR “increas* renal clearance” OR “enhanc* renal clearance” OR “enhance* renal function” OR “Renal hyperfiltration” OR “augmented kidney clearance”) AND (“vancomycin”). Records retrieved from all databases were compared, and duplicates were removed prior to the screening process.

### 2.2. Study Selection

All relevant studies that reported information on the impact of ARC on vancomycin therapy in critically ill adult patients were reviewed for inclusion. Studies that are duplicates, those in languages that are not easily translatable using online tools, non-human studies, pediatric populations, abstracts that were not yet published as full citations, as well as case reports, review articles, letters, opinion articles, and editorials were excluded. After removing duplicate records using the EndNote X9 software, titles and/or abstracts of all studies were reviewed for exclusion based on the eligibility criteria, and the full text was retrieved for potentially eligible studies. Any uncertainty regarding the eligibility or relevance of any of the articles was resolved through discussion among the authors. 

### 2.3. Data Extraction

The data were extracted independently by two investigators using a standardized form. For each article, the following data were collected: author name and year of publication, region and study period, study design, study purpose, study design, ARC definition, creatinine clearance (CrCl) determination method, population characteristics (age, sex, and setting), data on vancomycin administration (dose and frequency), and the main findings.

## 3. Results

### 3.1. Study Selection

**Figure 1:**
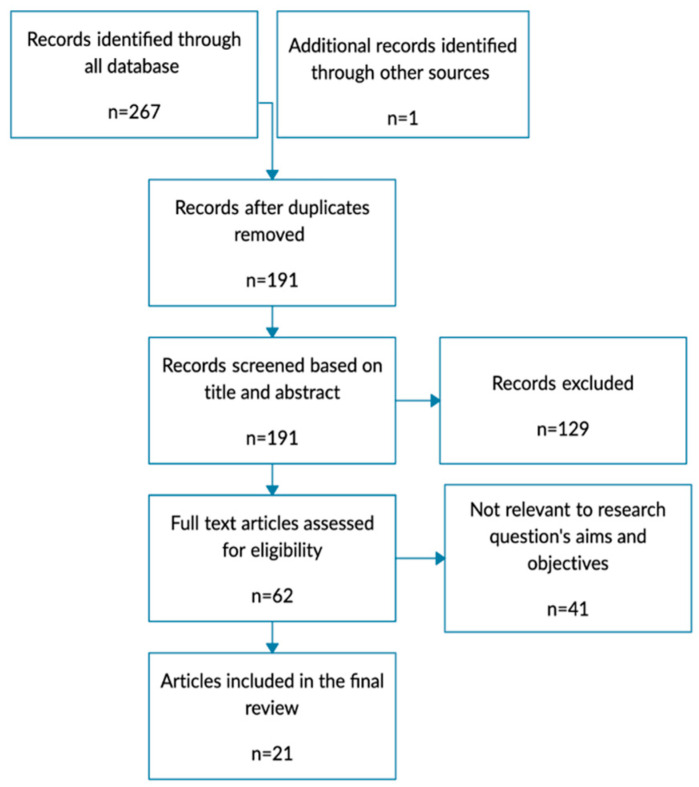
Flowchart of the results of literature search and selection process.

As described in Figure 1, a total of 267 records were initially collected from the databases, and one more recently published relevant article was identified through another source. After duplicate removal, 191 articles remained. Among these, 129 articles were excluded during the title and abstract review as they did not meet the selection criteria, resulting in 62 potentially relevant full-text articles. Following a full-text review, 21 articles met the inclusion criteria (Table S1). The primary reasons for exclusion were related to insufficient data on ARC or vancomycin, and studies focused on the pediatric population. 

Most of the included articles were observational studies, with 14 being retrospective and 5 prospective. One article [7] reported a combination of retrospective and prospective studies and the remaining article [12] was a randomized clinical trial. The studies included diverse ICU populations, including medical, surgical, neurosurgical, haemorrhagic stroke, traumatic brain injury (TBI), and septic patients, conducted in seven countries. While some studies did not specifically state the inclusion of critically ill patients or their specific diagnoses, they did include hospitalized patients with severe infections undergoing vancomycin therapy [18]. The age of participants in the studies varied from 33 to 76 years, and the proportion of male sex ranged from 28% to 80% across different populations (Table S1).

### 3.2. ARC Definition and Its Prevalence

Most of the recent studies defined ARC using the most recognized cut-off value of CrCl ≥ 130 mL/min, although different units were employed, such as mL/min and mL/min/1.73 m^2^. One exception was the study by Campassi M et al. [19], which used the cut-off value of ≥120 mL/min. Additionally, one study [20] did not report the specific definition of ARC but included a group of patients with CrCl ≥ 120 mL/min. The methods for determining CrCl varied among the studies. CG was the most frequently used CrCl estimation method in 12 studies (57%), followed by measured CrCl utilizing urine collection in five studies (24%), and the Chronic Kidney Disease Epidemiology Collaboration equation in one study (4%). Three studies [11,19,21] (14%) compared different methods for calculating CrCl, and one study [20] (4%) did not report the method used. 

In the vast majority of the included studies, critically ill patients were compared based on the presence or absence of ARC. ARC prevalence among these patients ranged from 16.4% to 72% [10,18,19,22,23,24,25,26,27]. The comparisons consistently revealed that younger age, male sex, heavier weight, lower illness severity, and the presence of brain injury or trauma were the factors most frequently linked with a higher risk for ARC. Other associated factors could be receiving mechanical ventilation, enteral nutrition, hemodynamic instability, low serum albumin, low platelet count, low serum creatinine, high glomerular filtration rate, presence of TBI, febrile neutropenia, trauma, intracerebral hemorrhage, and aneurysmal subarachnoid hemorrhage [20,23,26,28]. Furthermore, Zhao J et al. [26] conducted a study that aimed to evaluate two widely used scoring systems (ARCTIC and ARC risk scoring) to help define high ARC risk factors, revealing that 58.9% of ARC patients had high-risk scores when assessed in the ICU, while 88.9% had high-risk scores among trauma patients.

### 3.3. Impact of ARC on Vancomycin Therapy

In the reported studies, several population pharmacokinetic (PopPK) models have been established to predict dosing regimens and estimate vancomycin pharmacokinetics [8,18,27,29]. Some of the authors focused on developing new mathematical models and nomograms based on population PK and covariates to predict individualized vancomycin dosing regimens [7,28,30], while others used clinical data from TDM for dosage recommendation. Additionally, some PopPK software tools and nomogram validations were examined in patients with different renal functions [8,20].

The possible impact of ARC on the clinical outcomes of vancomycin was evaluated by most of the studies. This was based on evaluating the PK/PD indices. The most common PK/PD parameters reported were CL, V_d_, AUC_24_/MIC, and C_trough_. The main finding was decreased therapeutic concentrations following the increase in CrCl. When targeting a C_trough_ of 10–20 mg/L, the reviewed articles revealed that a significant proportion of patients with a CrCl ≥ 130 mL/min (ranging from 34–100%) experienced subtherapeutic trough vancomycin concentrations (C_trough_ < 10 mg/L) with standard vancomycin doses [7,8,10,12,18,20,22,23,24,25,26,31]. In a prospective study conducted by Campassi et al. that included 363 critically ill patients, ARC was assessed for its impact on serum vancomycin concentrations. The study found that, despite increasing doses of vancomycin, no patients with ARC achieved the target through concentrations [19]. ARC was further associated with subtherapeutic vancomycin trough concentrations in patients with hemorrhagic stroke, TBI, and those undergoing neurosurgery [25,29,32]. Similarly, studies assessing the impact of ARC on AUC_24_/MIC levels reported a consistent trend, with a higher rate of ARC patients falling below the recommended therapeutic targets compared to non-ARC patients. In a retrospective study conducted in a mixed ICU that included 280 vancomycin concentrations, it was reported that no ICU patients achieved the target AUC level of 400 mg.h/L. Moreover, patients with ARC exhibited a lower trend compared to the non-ARC group (232.9 vs. 316 mg.h/L) [24] (Table 1).

In a randomized clinical trial conducted by Sahrai et al., two different regimens of vancomycin administration (15 mg/kg every 12 h or 8 h) were compared in ARC patients, revealing that target AUC/MIC was achieved by a higher percentage of the group receiving vancomycin every eight hours compared to every 12 h (82.14% versus 46.42%) [12]. The reviewed studies collectively suggested that the typical dosage was insufficient and that increased dosing or dosing frequency is required to achieve adequate concentration; however, only a few studies provided specific dosing recommendations [12,24,30] (Table 2). 

## 4. Discussion

The main findings of the included studies suggest that higher doses of vancomycin may be necessary to reach therapeutic outcomes due to the enhanced drug clearance that is observed in patients with ARC.

### 4.1. The Critical Role of CrCl in Vancomycin Therapy and Identifying ARC

Effective antimicrobial therapy relies on appropriate dosing regimens, which, in turn, are determined by antimicrobial clearance, both crucial aspects for achieving a safe and therapeutic outcome [33]. Like many antimicrobials, vancomycin dosage is determined based on CrCl. CrCl is the volume of blood plasma cleared of creatinine, an endogenous filtration marker, per unit time and serves as an indicator of kidney function. CrCl can be directly measured from timed urine collection or estimated from serum creatinine (SCr) levels using equations such as CG and Chronic Kidney Disease Epidemiology Collaboration (CKD-EPI), with estimation being the more commonly applied method in clinical practice [34].

While there is no consensus on normal urine CrCl values, a CrCl above 130 mL/min/1.73 m^2^ is generally considered the standard lower limit for diagnosing ARC since it has been associated with subtherapeutic antimicrobial concentrations [35]. Elevated CrCl has been reported in patients admitted to the ICU, with an estimated ARC prevalence ranging from 30% to 65%, aligning with the reported ARC prevalence in the studies reviewed. While these findings are consistent with those reported in other studies, the variability in the range may be attributed to inconsistent methods for determining CrCl, variations in the definition of ARC, and diversity in the study population [6]. 

The CrCl estimating equations include factors such as age, sex, and body surface area in addition to SCr, serving as surrogates for muscle mass. Hence, they provide more useful data than SCr alone. However, these equations often exhibit a weak correlation with measured CrCl, particularly in critically ill patients with ARC [36]. These methods are developed through regression techniques to create a model that reflects the observed connection between the marker’s serum level and the measured glomerular filtration rate (GFR) within a specific study population. Nevertheless, their limited generalizability and the instability of SCr levels observed in ICU patients make these methods unreliable for estimating creatinine clearance and, thus, identifying ARC within these patient populations [34].

A recent prospective study compared both estimated (CG) and measured CrCl (8 h urine collection), revealing a higher CrCl with the 8 h urine collection method compared to the estimated one [26]. Similar findings were observed across the studies included, assessing the correlation between estimated CrCl using equations based on SCr and measured urinary CrCl using urine collection methods over defined time periods [11,19,29]. Additionally, Campassi M et al. reported a sensitivity of 39% for the estimated CrCl (CG) in diagnosing ARC [19]. More recently, another prospective, multicenter study conducted by Zhao J et al. examined the accuracy of CG estimates in predicting inadequate vancomycin PK/PD indices [26]. The accuracy for estimating C_trough_ and AUC_24_/MIC was 69.1% and 62.6%, respectively, indicating that a single CrCl estimate is a poor indicator for reaching the target values of PK/PD indices. The findings are not unexpected, given the interpatient variability in vancomycin exposure profiles observed in critically ill patients [15]. This presents a challenge in generating precise estimates of CrCl solely based on formulas derived from GFR estimation equations, potentially leading to overlooked cases of ARC and an underestimation of its prevalence. 

All the available data strongly support the use of measured CrCl as a more accurate method for assessing renal function. Among the specified time periods for urine collection, the 8-h-measured CrCl has consistently emerged as the most reliable indicator. Therefore, it is essential to utilize measured CrCl more frequently in clinical practice and consider alternative assessment tools such as ARCTIC and ARC risk scoring, which provide higher sensitivity and specificity in identifying patients at risk of ARC upon admission to the ICU [26].

### 4.2. Risk Factors for ARC 

The most consistently identified risk factor for ARC is younger age (<50 years old) [6,20,26]. It was reported as the most significant covariate in the majority of the included studies. These patients are generally less severely ill and exhibit a greater physiological reserve, contributing to a higher CrCl. Moreover, they tend to have higher CrCl due to the natural decrease in CrCl with age. Therefore, potential dose adjustments should be considered to mitigate the risk of underdosing in younger, healthier patients with elevated renal clearance. 

Another frequently reported risk factor associated with ARC in recent studies is heavier weight [8,18,25,26], given its impact on several physiological processes, including cardiac output and renal function [37]. Considering obesity as a major public health issue adds further significance to addressing and managing it in the context of vancomycin dosing and therapy for patients with ARC [26].

Furthermore, there is clear evidence that a higher prevalence of ARC has been increasingly associated with neurocritical patients, including those with traumatic brain injury (TBI) [10,32,36], recent history of trauma [6], central nervous system (CNS) infections [36], neurosurgery, and hemorrhagic stroke [29,32]. Although not fully understood, potential mechanisms that explain this relationship include the systemic inflammatory response syndrome (SIRS) resulting from activation of the immune system, a reduction in cerebral autoregulation, and an elevation in plasma concentration of the cardiac hormone atrial natriuretic peptide (ANP), often seen in TBI patients [6,36]. Further studies are required to comprehensively understand the pathophysiological mechanism between brain and kidney autoregulation.

### 4.3. Vancomycin Dosage Considerations 

The selection of an antimicrobial dosage regimen typically relies on the overall measure of the PK/PD parameters and specific effectiveness estimates, often quantified as MIC. Given that the bactericidal activity of vancomycin is primarily time-dependent, its efficacy relies on the duration of exposure to drug levels above the MIC to attain an optimal AUC_24_/MIC ratio, a PK/PD parameter that is closely correlated with its efficacy [36]. The optimal threshold for this vancomycin PK/PD efficacy is established as a target ratio of ≥400 mg.h/L [38]. In critically ill patients with normal renal function, an intravenous (IV) loading dose of 25–30 mg/kg followed by a maintenance dose of 15–20 mg/kg every 12 h a day is recommended to obtain adequate levels as soon as possible [15]. However, in critically ill patients with ARC, achieving sufficient vancomycin levels with standard doses becomes challenging due to the considerable PK alterations observed in this population. Furthermore, consistently maintaining low drug levels may increase the risk of MRSA resistance emergence, underscoring the crucial role of TDM for vancomycin to maximize its efficacy and decrease its nephrotoxicity [38].

The Infectious Disease Society of America (IDSA) previously recommended using vancomycin trough levels between 15 and 20 mg/L for severe MRSA infection and between 10 and 15 mg/L for non-severe MRSA infections to ensure an adequate AUC/MIC ratio of ≥400 mg.h/L [15]. However, relying solely on trough levels as a surrogate measure has limitations and is not well-correlated with AUC. This is due to the variability in AUC values resulting from the incorporation of peak concentrations, which depend on patients’ volume of distribution (V_d_). The recently updated guidelines suggest that using AUC for vancomycin dosing guidance provides superior clinical efficacy compared to the traditional approach of trough-based dosing. AUC_24_/MIC of 400–600 mg.h/L is recommended as the new PK/PD index, assuming vancomycin MIC at 1 mg/L [15]. The revised guideline proposes two approaches for estimating AUC-guided dosing. One involves the use of a first-order PK equation based on two drug concentrations measured 1–2 h after infusion and trough concentrations before the next dose. The other method employs population pharmacokinetic modeling (pop PK) along with Bayesian-derived AUC monitoring based on one or two concentrations, with at least one sample taken at the trough level [15]. In the present review, the development of a pop PK model to propose dosing recommendations has been supported by Zhao et al. [30], Chu Y et al. [28], and Yu XY et al. [8], owing to the software’s capacity to incorporate covariates such as age, body weight, and SCr, which were successfully estimated in the studies. Additionally, to adjust vancomycin dosing as early as possible, researchers have recommended performing early TDM, given the time-sensitive nature of the initial two days of MRSA treatment [27]. 

Vancomycin pharmacokinetics are best described by a two-compartment model because it provides a more precise prediction of drug concentrations [39], as supported by two reviewed studies [24,30]. However, some observational studies opted for a one-compartment model due to its mathematical simplicity and the limitation of retrospective TDM data obtained primarily based on C_trough_ [26,28,32]. Using a one-compartment model may introduce a significant bias when calculating AUC from PK profiles due to the model’s inability to characterize the area under the distribution phase [40]. Therefore, doses selected based on inaccurate estimations may fail to achieve the intended therapeutic outcomes. Further research is required to evaluate the appropriateness of the one-compartment model when predicting vancomycin exposure in special patient populations, such as those with ARC. 

### 4.4. Implication of ARC for Vancomycin PK/PD Indices

#### 4.4.1. AUC_24_/MIC and Trough Concentration

Despite the updated guidelines recommending the use of AUC for vancomycin dosage guidance, many institutions in clinical practice still rely on the use of steady-state trough concentrations (C_trough,ss_) for vancomycin dosing decisions. This is due to the challenges of obtaining multiple vancomycin levels needed for calculations and the common issue of poorly timed sample collection in clinical settings [41]. As a result, the older studies included in this review primarily assessed the impact of ARC on vancomycin C_trough_. Only a few recent studies have shifted focus to explore the association between ARC and AUC_24_/MIC indices. Collectively, these studies revealed a negative correlation between both vancomycin C_trough_ and AUC_24_/MIC with CrCl and, thus, vancomycin clearance in ARC patients. It was consistently observed that the conventional administration of vancomycin did not meet the desired PK/PD targets for ARC patients (Table 1). Notably, some studies revealed persistently low trough levels (<10 mg/L) even with higher dosages in ARC patients [10,19,26]. However, high vancomycin exposure can lead to toxicity such as acute kidney injury (AKI) [42], and clinicians should be mindful of this potential risk when adjusting vancomycin dosage for ARC patients. 

#### 4.4.2. CL, Half-Life, and V_d_

Lower SCr has been consistently identified as an independent risk factor for ARC [7,24,31]. A recent study conducted in China [8] revealed a significant correlation between SCr levels and vancomycin clearance, suggesting that substantial changes in patients’ creatinine levels during and after vancomycin treatment can directly impact drug clearance. The study reported a shift of more than 50% in SCr for patients with varying renal function, resulting in lower SCr for patients with ARC due to the elevated CrCl. The implications in terms of enhanced drug elimination are significant for these patients, consequently leading to a shorter drug half-life and significantly lower AUC and subtherapeutic concentration compared to patients with normal renal function. The reviewed Pop PK studies confirmed the trend of elevated CL, reporting vancomycin clearance reaching levels 1.3–3.5 times higher in patients with ARC compared to those without ARC [8,24,28,30] (Table 1).

A larger volume of distribution of hydrophilic drugs has been observed in ICU patients, possibly attributed to the increased blood flow to major organs resulting from hyperdynamic status and increased cardiac output in this patient population. A recently published Pop PK study reported a more than threefold increase in the central volume of distribution in ICU patients compared to non-ICU patients [28,30] (Table 1). However, its clinical relevance to vancomycin dosage is unclear, as the AUC primarily depends on the clearance rate during steady state [30]. On the contrary, in patients with ARC, vancomycin may distribute extensively in the body due its higher V_d_, potentially reaching infection sites that were less accessible before. This characteristic can be strategically utilized to optimize loading doses, potentially enhancing the body’s exposure to vancomycin during the initial treatment phase for patients with ARC [30].

The observed vancomycin PK/PD alterations in critically ill patients with ARC carry significant implications for patient outcomes. Therefore, it is important to comprehensively understand these PK/PD alterations and identify optimal PK/PD targets to ensure the efficacy of vancomycin therapy.

### 4.5. Approach to Vancomycin Dosing in Patients with ARC

In critically ill patients, particularly those with ARC, achieving effective vancomycin plasma concentration rapidly is crucial to avoid subtherapeutic outcomes and potential adverse effects. A recent retrospective study, including 141 critically ill patients treated with vancomycin, evaluated the AUC on days 1 and 2 and at steady state using the probability of target attainment (PTA) based on Bayesian estimation [27]. The study revealed that the AUC at TDM was significantly higher than AUC at the initial dose design; therefore, early TDM is essential for adjusting individual doses of vancomycin in patients with ARC, ensuring adequate drug exposure, and preventing overdosages. 

Given the high prevalence of ARC in ICU patients, routine screening for ARC in this setting is recommended. While the available ARC screening systems serve as useful initial screening tools for ARC, additional risk factors associated with a higher ARC incidence, such as TBI, SAH, and neurosurgery, should be taken into account. Patients identified as at high risk for ARC should undergo an 8 h continuous urine collection for CrCl determination. If the measured CrCl is ≥130 mL/min, a higher dose is necessary for similar drug exposure during the initial dosing. Suggested dosing recommendations for ARC patients are outlined in Table 2, drawing from the current literature reviewed in this study.

For the initial vancomycin regimen in critically ill patients with ARC, it is advised to consider a loading dose of 30 mg/kg. While certain studies indicate that a loading dose may not increase the risk of AKI, caution is warranted when considering loading doses exceeding 3 g [43]. This review additionally recommends maintenance doses of 15–20 mg/kg q 8 h, however, careful consideration should be given to daily doses exceeding 4 g to minimize adverse effects. Individualized dosing guided by TDM results is recommended for dose readjustments based on CrCl.

There are some limitations to this review. Firstly, given the challenges of conducting clinical research in the ICU setting, where a higher mortality rate and unpredictable patient status are prevalent, the majority of included studies were observational, single-center studies leading to a lack of high-quality data. To establish more robust evidence for vancomycin dosing strategies in patients with ARC, there is a clear need for larger samples or multicenter prospective studies. Secondly, the review utilized only two databases for the literature search, possibly limiting the inclusion of all relevant studies and thereby missing valuable information from sources beyond the scope of the selected databases. Nevertheless, this review provides valuable insights into the main features of ARC and vancomycin therapy, serving as a resource to inspire both researchers and clinicians in understanding and addressing the discussed challenges, which should be further evaluated in future studies.

ARC greatly affects and impacts vancomycin PK/PD, so it is essential to consider a multifaceted approach to optimize therapeutic outcomes. In severe infections such as sepsis, each one-hour delay in the administration of antibiotics could lead to a 9% increase in mortality [44]. From our perspective, understanding the effect of ARC and its impact on vancomycin PK/PD is paramount to achieving effective dosing strategies, especially in critically ill adult patients. It is important to tailor dosing strategies that account for individual characteristics, such as renal function, to minimize vancomycin toxicity and maximize therapeutic efficacy.

## 5. Conclusions

This review consolidates what is currently known regarding vancomycin therapy in ICU patients with ARC and identifies important gaps for future research. The consistent recommendation for upward dosage adjustments in the reviewed studies underscores the need for clearer guidelines within the ICU setting. Considering the observed vancomycin subtherapeutic concentrations associated with ARC, there is an urgent need for reliable methods to assess renal function, as early identification of ARC is crucial for the effective management of vancomycin treatment. As data from a future perspective and multicenter interventional clinical studies emerge, collaborative efforts among clinicians and researchers become essential to effectively address and resolve these challenges and establish individualized vancomycin dosing guidelines for critically ill patients experiencing ARC. 

## Figures and Tables

**Table 1:** Overview of vancomycin PK/PD indices in patients with ARC.

Population	Age ^ab^ (Years)	CrCl ^ab^ *	Maintenance Dose ^ab^ **	C_trough_ (mg/L) ^ab^	C_trough_ < 10 mg/L, (%)	AUC_24_ (mg.h/L) ^ab^	V_d_ (L) ^ab^	VCM CL (L/h) ^ab^	References
Mixed ICU	69 (59–75)	160.3 (144.2–199.9)	14.7 (13.0–18.2)	NR	NR	240 (209–300)	NR	NR	Ishigo T et al., 2023 [27]
Mixed ICU	69 (50–73)	171.6 (157.5–203.0) mL/min	34.2 (28.3–42.1)	9.4 (5.9–11.9)	NR	NR	NR	NR	Mikami R et al., 2022 [11]
Mixed ICU	BD: 44.04 ± 16.55 TDS: 42.86 ± 11.83	BD: 166.94 ± 41.32 TDS: 171.78 ± 48.56	15 mg/kg	BD: 5.64 ± 1.92 TDS: 14.03 ± 2.97	NR	BD: 397.90 ± 76.02 TDS: 611.92 ± 148.01	BD: 44.39 ± 14.21 TDS: 41.87 ± 27.30	BD: 5.97 ± 1.48 TDS: 5.69 ± 1.87	Sahraei Z et al., 2022 [12]
ICU and non-ICU	50.9 ± 15.1	141.2 ± 16.0	30.3 ± 6.4 mg/kg	7.1 ± 2.9 mg/mL	80	JPKD: 307.4 ± 72.4 SDose: 376.6 ± 103.4	JPKD: 72.6 ± 10.3 SDose: 44.6 ± 6.7	NR	Yu XY et al., 2022 [8]
ICU and non-ICU	50 (33–60)	159 (144–193)	2 g/day	7.1 (3.9–10.6)	71.6	(253.8–475.0)	NR	NR	Zhao J et al., 2022 [26]
ICU	33 (26–46)	168.4 (148.5–193.2)	1.28 ± 0.52 g	6.45 (3.72–8.64)	80.77	NR	NR	NR	Chen Y et al., 2020 [25]
Hospitalized	45 (33–57.25)	180.50 (152.95–207.35) mL/min	1000 mg every 12 h	6.80 (3.50–13.30)	>60	NR	NR	NR	Chu Y et al., 2020 [18]
Hospitalaized	45 (33–57.25)	175.90 (142.20–198.10) mL/min	1000–4000 mg/d every 6, 8 and 12 h	NR	NR	NR	155.4	8.52	Chu Y et al., 2020 [28]
Mixed ICU	40.0 ±11	180.8 ± 59.3 mL/min	29 ± 9.4	6.5 ± 3.8	77.7	232.9 ± 93.6	69.3 ± 9.1	9.7 ± 3.4	He J et al., 2020 [24]
ICH and aSAH	63.3 ± 13.3	161.6 ± 16.7 mL/min	15.1 ± 4.2 every 8 h (8–12)	12 ± 3.6	NR	NR	71.8 ± 11.3	NR	Morbitzer KA et al., 2019 [29]
Adult patients	43.8 ± 15.9	187.7 ± 50.0	1000 mg every 8 h	NR	62.9	NR	NR	NR	Chu Y et al., 2016 [31]
Mixed ICU	57.5 (39.0–69.3)	157.4 (142.1–173.9)	35.7 (30.5–40.0)	7.4 (5.2–11.6)	NR	447 (400–554)	133 (112–147)	5.3 (4.9–6.02)	Hirai K et al., 2016 [10]
Mixed ICU	48±15	155±33	30	NR	100	NR	NR	NR	Campassi M et al., 2014 [19]
ICU and non-ICU	45.5 (21–66)	150.5 (42); 131–324	<15 15–30 >30	NS	31.8	NR	NR	NR	Minkute R et al., 2013 [23]
ICU	41 (32–56)	158.9 (140.9–193.6)	30 (25.0–32.3) mg/kg	D1: 14 D3: 20	D1: (98.2) D3: (48)	NR	NR	NR	Baptista JP et al., 2012 [22]

^a^ Data is presented as median (interquartile range) or ^b^ mean ± standard deviation (SD). * CrCl is reported in mL/min/1.73 m^2^ and ** maintenance dose is reported in mg/kg/day unless indicated otherwise. Abbreviations; aSAH, aneurysmal subarachnoid hemorrhage; AUC_24_, area under the plasma concentration-time curve over on day 1; BD, every 12 h group; CL, clearance; CrCl, creatinine clearance; C_trough_, trough concentration; D, day; ICH, intracerebral hemorrhage; ICU, intensive care unit; JPKD, JavaPK for Desktop; NR, not reported; q, dose frequency; SDose, SmartDose; TDS, every 8 h group; V_d_, volume of distribution; VCM, Vancomycin.

**Table 2:** Proposed vancomycin dosing recommendations in patients with ARC.

CrCl (mL/min)	Dosage Regimen	PTA (%)	PD Target	Based on	References
120–149	1750 mg q24 h	62.33	AUC_24_ (400–650 mg.h/L)	PopPK study (Model-based Monte Carlo Simulations)	Zhao S et al., 2021 [30]
150–179	1000 mg q12 h	62.56			
≥180	750 mg q8 h	61.69			
≥130	46 mg/kg/day		Ct_rough_ > 10 mg/L	PopPK study (Bayesian estimation)	He J et al., 2020 [24]
	69 mg/kg/day *		C_trough_ > 15 mg/L *		
≥130	15 mg/kg q8h		AUC/MIC > 400	RCT	Sahraei Z et al., 2022 [12]

* In severe cases. CrCl, creatinine clearance; PopPK, population pharmacokinetics; PTA, probability of target attainment; RCT, randomized clinical study; q, dose frequency.

## Data Availability

All relevant data are within the manuscript.

## Supplementary Materials

The following supporting information can be downloaded at: https://www.mdpi.com/article/10.3390/jcm13082317/s1, Table S1: Summary of the studies included in the current review. References [7,8,10,11,12,18,19,20,21,22,23,24,25,26,27,28,30,31,32,45,46] are cited in the Supplementary Materials.
