# Supplementary material for: The Impact of Augmented Renal Clearance on Vancomycin Pharmacokinetics and Pharmacodynamics in Critically Ill Patients
Source: J Clin Med. 2024 Apr 17;13(8):2317. doi: 10.3390/jcm13082317 (PMC11051385; doi:10.3390/jcm13082317)
Supplement: Supplementary file 1 [file jcm-13-02317-s001.zip › jcm-2946726-supplementary.pdf]

Author, year	Region, study period	Study purpose	Study design	Population	N	Age (years) Median (IQR), Mean ± SD	Sex n (% male)	ARCdefinition (mL/min/1.73 m²) [a]	CrCl determination method	Vancomycin dosage regimen	Key findings
Ishigo T et al. 2023 [1]4/17/2024 3:26:00 PM	Japan (January 2020 – December 2022)	- To explore factors linked with the attainment of target AUC during follow-up<br><br>- Provide a decision flowchart for achieving target AUC	R (multi-center)	Mixed ICU	70	66 (56, 79)	35(50)	≥ 130	Calculated CG	LD : 25.4 (21.4, 29.1) mg/kg<br>MD: 26.5 (19.7, 34.2) mg/kg/day	• The prevalence of ARC was 15.7%. During follow-up, 70% of 70 met the target AUC, while the target was reached by only 45% of ARC patients.<br>• The number of VCM administered doses was significantly higher in those achieving the target AUC**, though, this significance vanished when considering only predicted AUC target values<br>• A decisional flowchart was developed based on achieving target AUC, dosing interval, and the CrCl.
Ishigo T et al. 2023 [2]	Japan (January 2018-April 2022)	To study the impact of ARC on early (day -1 and -2) AUCs deviations (defined as AUC deviation of ≥ 30% upward at TDM from AUC at initial dosing) of VCM	R (single-center)	Mixed ICU	141<br>ARC: 35<br>non - ARC:106	ARC: 69(59-75)<br>non-ARC: 58 (47-63)	ARC: 23 (65.7) non ARC: 76 (71.7)	≥ 130	Calculated CG	ID: 20.7 (16.3, 24.7) mg/kg<br>MD: 14.3 (11.7, 16.7) mg/kg	• Initial simulations compared to TDM revealed a significant increase in AUC_{24} in ARC group (± SD:75.9 ± 112.8)*.<br>• During the TDM period, AUC_{0-24}, AUC_{24-48}, and AUC_{ss} were significantly lower in ARC compared to the non-ARC (AUC_{ss}: 338 [244-486] vs 504 [392-600] mg.h/L).<br>• AUC deviations were higher in the ARC group (57%) compared to the non-ARC group (16%)*.<br>• Patients in the ARC group were younger, had higher BMI and higher catecholamine use.
Mikami R et al. 2022 [3]	Japan (April 2014 – July 2020)	- To compare CrCl estimating methods and their correlation with VCM C_{trough}/ MD.<br>- To compare VCM C_{trough}/ MD between ARC and non-ARC patients	R (single-center)	Mixed ICU	Actual ARC (14),<br>Borderline (130 mL/min (at the start and < 130 mL/min after that) (8)<br>non-ARC (43)	69 (50-73)	45(69.2)	> 130 mL/min	1) Measured (8-h)<br>2) Calculated (CG)<br>3) Calculated (KeGFR).	LD: (100-2000 mg)<br>MD: mg/kg/day<br>ARC: 34.2 (28.3-42.1)<br>Borderline: 42.3 (18.2-57.3)<br>non ARC: 29.4 (17.9-32.0)	• Measured CrCl demonstrated the strongest correlation with VCM C/D ratio (r=-0.598)*, surpassing the calculated (KeGFR) (r = - 0.422) and CG (r = -0.426). However, none of the methods showed a significant correlation with non-ARC patients experiencing a change in renal function.<br>• The VCM C/D ratio was significantly lower in patients with the ARC group (0.24 kg/L) compared to non-ARC patients (0.52 kg/L)*.

Sahraei Z et al.<br>2022 [4]	Iran (April 2021 – June 2022)	To compare VCM pK parameters in ARC patients between two different treatment regimens, BD(q12 h) and TDS (q8 h)	RCT	Mixed ICU	56<br><br>BD (q 12 h): 28<br>TDS (q 8 h): 28	BD: 44.04 ± 16.55<br>TDS: 42.86 ± 11.83	BD: 17 (60)<br>TDS:16 (57)	≥ 130 mL/min	Measured (8 h)	LD: 20mg/kg<br>MD: 15 mg/kg	• AUC, C_{trough}, and peak concentrations were significantly lower in BD than in the TDS group.<br>• At a MIC of 1 mcg/ml, a higher percentage of TDS achieved the target AUC/MIC compared to BD (82.14% vs 46.42%, p = 0.006).<br>• Despite these differences, Ke, $t_{1/2}$, CL, and V_d showed no significant variation between the two groups
Yu XY et al.<br>2022 [5]	China (May 2014 to December 2019)	To assess the predictive accuracy of PPK software programs (JPKD and SmartDose) in estimating VCM Pk in patients with different renal function	R (single-center)	ICU and non-ICU	388<br><br>ARC : 86<br>NFR (60 ≤ CrCl ≤ 130): 241<br>IRF: (CrCl < 60): 61	ARC: 50.9 ± 15.1<br>NRF: 68.5 ± 13.4<br>IRF: 76.6 ± 13.2	ARC: 62 (72.1)<br>NRF: 116 (48.1)<br>IRF: 36 (59)	≥ 130	Calculated (CKD-EPI)	Initial regimen (mg/kg)<br>ARC: 30.3 ± 6.4<br>NRF: 31.7 ± 8.1<br>Adjusted regimen: (mg/kg)<br>ARC: 39.4 ± 9.7<br>NRF 33.9 ± 9.8	• Younger, taller, and heavier males were more prevalent in ARC patients.ARC correlated positively with postoperative and IRF (P= 0.028, P= 0.012) and negatively with CI, diabetes, and heart failure (P= 0.019, P= 0.028. P= 0.015).<br>• 8.1% in ARC, 17.8% in NRF and 31.1 % in IRF experienced a more than 50% change in their creatinine levels post VCM treatment:<br>• Despite the initial and adjusted total daily doses being higher in ARC than NFR*, C_{trough} levels were lower in ARC, with a higher percentage (84.9%) of ARC patients having C_{trough} < 10 mg/L.<br>• JPKD showed better precision in predicting C_{trough} for the adjusted regimen
Zhao J et al.<br>2022 [6]	China (September 2012 - July 2020)	- To explore risk factors linked to ARC and its impact of PK/PD of VCM.<br><br>- To assesses the efficacy of two scoring systems as screening tools to identify high ARC risk (defined as ARC score ≥ 7 and ARCTIC score ≥ 6)	P (multi-center)	ICU and non-ICU	414<br><br>ARC: 88<br>non ARC: 326	ARC: 50 (33-60)<br>non ARC: 64 (53-76)	ARC: 66 (75)<br>non ARC: 211 (64.7)	≥ 130	Calculated (CG)	Initial daily dose (g/day):<br>ARC: 2 (2-2)<br>Non-ARC: 2 (1-2)	• Positive correlations with ARC included, male sex, age < 50 years, overweight status, mechanical ventilation, and enteral nutrition while cardiovascular disease and higher neutrophil count were negative correlates.<br>• ARC patients more frequently fell below recommended targets for trough levels and AUC_{24}/MIC compared to non ARC patients (p =0.003).<br>• Despite higher initial doses in ARC group, PK/PD indices had significantly lower values, negatively correlating with CrCl. Even when adjusted for dose, ARC levels

											remained significantly lower than non ARC*.<br>• Estimated CrCl was ineffective in predicting PK/PD values.<br>• A high ARC risk score predicted subtherapeutic C_{trough} and AUC_{24}/, while the ARCTIC high specifically predicted subtherapeutic AUC24/MIC, (p = 0.013)
Zhao S et al. 2021 [7]	China (January 2010 – June 2018)	Pop PK study of VCM to recommend dosage for patients with different renal function including ARC	R (single-center)	ICU and non - ICU	209	66.0 ± 16.4	126 (60.3)	≥ 130 mL/min	Calculated (CG)	1875.0 (1461.9 - 2352.0) mg/day	• ARC incidence was 24%.<br>• A Pop PK model with two compartments was the best fit for VCM PK profile and a final model equation was formulated to represent the concentration of VCM change over itme.<br>• The model evaluation indicated that the final model predictions agreed with the observed plasma concentrations of VCM.<br>• Various dosing regimens were caclulated using the final model to assess their probability of achieving the desired target AUC_{24} based on VCM CL and V_d.<br>• The dose of VCM increased with increasing CrCl for patients with CrCl ≤ 180 mL/min. Patients with a CrCl ≥ 180 mL/min did not need to increase the dose.

Chen Y et al. 2020 [8]	China (January 2019 – June 2019)	To study the impact of ARC on vancomycin TDM in patients undergoing neurosurgery	R (single center)	Neurosurgical	N=104<br><br>ARC: 26<br>non ARC: 78	ARC: 33 (26,46)<br>non ARC: 56 (45, 62)	ARC: 134 (72)<br><br>non ARC: 53/25 of 78 male	≥ 130	Calculated (CG)	ARC: 1.28 ± 0.52 g<br>non-ARC: 0.87 ± 0.43 g	• Younger age, heavier weight and higher CrCl were identified as risk factors for ARC**.<br>• The mean C_{trough} were significantly lower in the ARC group 6.45 mg/L (3.72, 8.64) compared to the non-ARC group 10.72 mg/L (6.97, 16.55)**, with fewer ARC patients achieving the target C_{trough} (19.3% vs 40.03%).<br>• C_{trough} was correlated positively with age (r = 0.236, P=0.017), while correlating negatively with CrCl (r = -0.281, P = 0.004).
Chu Y et al. 2020 [9]	China (May 2013 to October 2016)	To analyse factors affecting the C_{trough} of VCM in patients with ARC	R (single-center)	Hospitalized	ARC: 186<br>non ARC: 69	ARC: 45 (33 - 57.25)<br>non ARC: 56 (49-63)	ARC: 134 (72)<br><br>non-ARC: 69 (57)	≥ 130 mL/min	Calculated (CG)	1000 mg q 12 h	• Younger age, heavier weight, higher CrCl and lower serum albumin, platelets count, and trough concentrations were identified as risk factors for ARC**.<br>• More than 60% of ARC patients had a C_{trough} of < 10 mg/L while only 2.69% had a Ctrough of > 20mg/L.<br>• After subgrouping ARC patients into three groups based on the CrCl, it was found that age and CrCl were the main factors that caused a statistically significant difference in the C_{trough} between the groups**. < 40 years: Ctrough: 5.90 (2.70-13.88)
Chu Y et al. 2020 [10]	China (July 2013 – December 2015)	A PopPk analysis was performed to provide a good model for VCM C_{trough} prediction for ARC patients based on population PK parameters.	R (single-center)	Hospitalized	95	45.00 (30.00–57.00)	71(74.7)	≥ 130 mL/min	Calculated (CG)	(q6h, q8h or q12 h) with a daily dosage of 1000 –4000 mg	• A robust and reliable PopPK model was developed for predicting individual VCM PK parameters in ARC patients.<br>• A one-compartment model was used to estimate the population parameters.<br>• The age was identified as the most significant covariate in the final PopPK model.<br>• CL and V_d in the final model were 8.52 L/h and 155.4 L.

Helset E et al. 2020 [11]	Norway (May 2013 – October 2015)	To observe the difference between CRRT and non-CRRT in achieving the target AUC_{24}/MIC ratio of >400.	P (single-center)	Mixed ICU	83 Non CRRT: ARC (21) non - ARC group (22)	54.5 (38-63)	61 (73.5)	≥ 130 mL/min	measured (24h)	MD: ARC: 44.4 (28.7-60.1) and non-ARC: 29.6 (21.3 - 38.0) mg/kg	• The AUC_{24}/MIC ratio was higher in the CRRT group compared to the non-CRRT group*.<br>• Significant differences in vancomycin AUC_{24}/MIC ratio were observed between the ARC group and the other groups, with the ARC group demonstrating the lowest ratio**
He J et al. 2020 [12]	China (Januray 2013 – November 2018)	To determine the PK/PD of VCM and recommend an optimal dosage regimen for critically ill patients with ARC	R (single-center)	Mixed ICU	ARC: 139 non ARC: 141	ARC: 40.0 ±11<br><br>non-ARC: 55.0 ±11.0	ARC:90(64.7)<br><br>non-ARC:75 (53.2)	≥ 130 mL/min	Calculated (CG)	Initial dose: 15mg/kg q 12 h	• Male patients, younger age and higher CrCl were more prevalent in ARC group**.<br>• All ICU patients had AUC24 values below the recommended target. The ARC group had an even lower AUC24 value (232.9 μg·h/mL) compared to the non-ARC group (316 μg·h/mL)<br>• 77.7% of ARC patients and 68.8% of the non-ARC patients had C_{trough} < 10 mg/L**<br>• To achieve a target C_{trough} of 10 mg/L, a daily dose of 46.0 and 35.5 mg/kg is recommended for ARC and non-ARC group, respectively
Morbitzer KA et al. 2019 [13]	USA (NR)	To determine if patients with hemorrhagic stroke experiencing ARC exhibited alterations in VCM PK parameters compared to those predicted by Pop PK	P (single-center)	Adult patients with ICH or aSAH admitted to the neurosciences intensive care unit	17	63.3 ± 13.3	6(35)	≥ 130	Calculated (CG) and measured (8 h)	15.1 ± 4.2 mg/kg q 8 h	• The measured CrCl was significantly higher than the estimated (161.6 ± 16.7 vs 116.7 ± 10.3 mL/min)*.<br>• 71% of patients experienced ARC, however, all the patients had enhanced CrCl on the day of vancomycin concentration measurements.<br>• The calculated k_e and consequently the $t_{1/2}$ were significantly lower in the predicated values based on population data compared to the measured values**. On the other hand, there was no significant change in the V_d between measured and predicted values. These alterations resulted in C_{trough} lower than predicted (10.3 ± 4.3 vs 18.3 ± 8.6 ug/mL)
Nelson NR et al. 2019 [14]	USA (April 2014 – December 2015)	To compare PopPk-based parameters using capping CrCl at 120	R (single-center)	Adult patients with TBI	32	36 (24.5-52)	23(72)	≥ 130	Calculated (CG)	17.1 (13.2–19.2) mg/ kg every 8 h	• 75% of TBI patients had ARC.<br>• There were no significant differences found between predicted and patient-specific measurments of k_e and C_{trough} when using the non-capped CrCl**.

		mL/min/1.73 and non-capping CrCl when determining VCM doses in patients with TBI									• Using the capped CrCl at 120 mL/min resulted in a significantly lower k_e [0.104 (0.104–0.104) vs. 0.13 (0.12–0.15)] and C_{trough} [16.3 (15.3–22.0) vs. 11.5 (7.8–13.7) mcg/mL] between predicted and measured values respectively*.
Ishii H et al. 2018 [15]	Japan (January 2013 - December 2017)	To validate the nomogram used for dosing VCM in patients with ARC and to investigate how specific clinical conditions associated with ARC might affect its accuracy	R (single-center)	Adult patients receiving IV VCM	nomogram dosing: 177<br>age < 50: 23<br>age ≥ 50: 154	73 (63-80)	109 (62)	NR	NR	29.0 (16.8-35.6) mg/kg/day	• Age was the sole factor linked to subtherapeutic C_{trough}, with patients < 50 having lower levels (7.3 [5.2 - 9.9]) than those 50 and older (11.9[7.9-14.5]mcg/mL), (p = 0.001) while no significant difference were found in C_{trough} based on CrCl.<br>• Only 22% of patients < 50 reached target C_{trough} compared to 44 % in the 50 and older group, with 74% having levels < 10 mg/L,(p = 0.014).<br>• The nomogram dosing method achieved a 47% accuracy rate in attaining the desired C_{trough}, with only 7% exceeding the target.<br>• Specific patient conditions, like febrile neutropenia, solid tumor, and blood cancer were associated with a significant increase in VCM CL (p = 0.003, p = 0.026, and p = 0.017 respectively), these effects remained significant even after adjusting for age. On the contrary, VCM C_{trough} showed no significant association with these patient conditions.
Chu Y et al. 2016 [16]	China (May 2013 - May 2015)	To evaluate the impact of ARC on serum VCM concentration	R (single-center)	Adult patients treated with VCM	148<br>Group A: CrCl < 80 mL/min (42)<br>Group B: 80 ≤ CrCl < 130 mL/min (36)<br>Group C : ≥ 130 mL/min (70)	Group A 63.3 ± 15.1<br>Group B 58.9 ± 13.6<br>Group C 43.8 ± 15.9	97(66)	≥ 130 mL/min	Calculated (CG)	Initial dose: 1000 mg every 12 h<br>Adjusted dose for ARC: 1000mg every 8 h and 1500 mg every 12 h	• Younger age, low SCr, and GFR were the main factors associated with ARC*.<br>• Around 69.2% of the ARC group didn't achieve the target C_{trough}<br>• VCM C_{trough} was negatively correlated with the CrCl (R^2=0.699, R^2=0.488)* and it was significantly lower in the ARC group despite the same dosage regimen.<br>• After dose adjustment to 1000mg every 8h, 2 patients increased from 3.7 to 5.2 ug/mL and 10.2 to 12.1 ug/mL.

											• When the dose was adjusted to 1500 mg every 12 h, only one patient increased from 4.8 to 8.2 ug/mL, which was still lower than the target C_{trough}.
Hirai K et al.<br>2016 [17]	Japan (April 2013 - February 2016	To identify the risk factors associated with ARC and to assess the impact of ARC on VCM PK parameters	retrospective observational	ICU and non-ICU	n: 292<br>ARC: 48<br>non ARC: 244	ARC: 57.5 (39.0 - 69.3)<br>non ARC: 74.0 (65.0 - 83.0)	ARC: 30 (62.5) non ARC: 155 (63.5)	≥ 130	Calculated (CG)	ARC: 35.7 (30.5-40.0)<br>non ARC: 27.1 (19.3 - 36.6) mg/kg/day	• The prevalence of ARC was 16.4%.<br>• Younger age (≤ 60 years), presence of brain injury and febrile neutropenia, and a volume of infusion fluid ≥ 1500 mL/day were identified as independent risk factors for ARC.<br>• There was a significant correlation between CrCl and VCM CL in ARC patients (r = 0.8726, P = 0.0001).<br>• ARC patients had higher CL VCM values compared to non-ARC patients<br>• Despite higher doses, the ARC group exhibited lower C_{trough} (7.4 [5.2 - 11.6] vs 12.2 [8.9 - 16.3], p < 0.0001) and AUC (447 [400-554] vs 554 [442-720], p = 0007).<br>• A higher percentage of patients with ARC (68.8%) had C_{trough} below the desired target compared to the non-ARC (32.8) (p < 0.0001)
Baptista JP et al.<br>2014 [18]	Portugal (over 13 months)	To develop a dosing nomogram for VCM and evaluate its efficiency in critically ill patients.	R (single-center) followed by P (single-center)	Sepsis ICU	104<br><br>Group1 retrospective cohort (79)<br><br>Group 2 prospective cohort (nomogram dosing) (25)	Group 1: 57.8 (15.5)<br>Group 2: 59.9 (17.2)	Group 1: 52 (66.0)<br>Group 2: 17 (68.0)	>130	Measured (8h)	LD: group 1: 14.3 (12.8 - 17.6) mg/kg group 2: 18.8 (16.7-21.4)<br><br>Total dose: group 1: 3160 (2520-3880) mg/day<br>group 2: 3584 (2976-4138)	• The incidence of ARC was 36% and 40% in groups 1 and 2 respectively.<br>• Group 2 had a significantly higher rate of target VCM serum concentration attainment (84%) compared to group 1 (51%)**<br>• There was a significant correlation between CrCl and VCM CL on day 1 (r^2 = 0.66)*.<br>• After applying the proposed nomogram all ARC patients in group 2 achieved desired therapeutic concentrations, whereas only 28% of ARC patients in group 1 were able to achieve the target concentration.<br>• VCM serum concentrations between 20 to 30 mg/L were considered adequate.
Campassi M et al.<br>2014 [19]	NR (October 2011 - September 2012)	To investigate the incidence of ARC and its impact on vancomycin	P (single-center)	Mixed medical-surgical ICU	n: 363<br>44 (received VCM)	ARC 48±15<br>non- ARC 65±17	103(28.4)	≥ 120	A comparison of the measured (24) vs Calculated (CG)	LD: 15 mg/kg<br>MD: 30 mg/kg/day	• 103 patients (28%) experienced ARC.<br>• Patients with ARC were younger, were more frequently admitted for obstetric and trauma-related reasons, had lower APACHE II scores, had

		concentrations in ICU patients									larger urine volume and higher elimination of electrolytes compared to non-ARC patients**<br>• While the estimated and the 24 CrCl were significantly correlated, they did not match very closely, with the accuracy and specificity of the estimated for identifying ARC being 39.8% and 90.8%, respectively<br>• 24 h CrCl was positively correlated with age ($R^2 = 0.28$) * and negatively correlated with VCM concentrations ($R^2 = -0.26$, $P<0.0001$),<br>• Regardless of increasing the dose, none of the ARC patients reached the target concentrations
Minkute R et al. 2013 [20]	Lithuania (2010 - 2011)	To estimate the prevalence of ARC and its effect on VCM trough concentrations by employing VSCss for analysis	R (single-center)	Patients who had measured VSCss	36	ARC 45.5 (15); 21–66 non-ARC 54 (27); 22–86	29(80)	≥ 130	Calculated (CG)	1000 -4000 mg/day	• ARC had a prevalence of 50%<br>• Younger age, mechanical ventilation, and hemodynamic instability were significantly predominant in ARC patients**.<br>• Within the ARC group, 34.2% did not achieve therapeutic VCM concentrations and had significantly lower C_{trough} despite higher doses**.<br>• A larger deviation in the subtherapeutic concentration due to the distribution of doses within groups was also observed in the ARC group.<br>• Different cutoff values for estimated CrCl (CG) were analyzed to predict trough VSC and a negative correlation was observed, particularly strong when CrCl≥ 150 mL/min ($r^2 = 0581$; $r^2 = 0224$; $P = 0005$).<br>• Gender and vancomycin dose were identified as the two most important factors in the prediction model for trough VSCss in patients with ARC

Baptista JP et al. 2012 [21]	Portugal (March 2006 - February 2008.)	To investigate the effect of ARC on VCM serum concentrations in ICU patients	P (single-center)	Sepsis ICU	93<br>ARC: 37<br>Non - ARC: 56	ARC 41(32–56)<br>non- ARC 70 (52–79)	ARC: 40 (71.4)<br>non-ARC: 29 (78.4)	> 130	Measured (24)	LD: ARC: 1.0 (1.0-1.5) g<br>non- ARC : 1.0 (1.0 - 1.1) g<br><br>Perfusion dose: ARC: 30 (25.0 - 32.2)mg/kg<br>non-ARC: 30 (26.7-34.4) mg/kg	• ARC was prevalent in 40% of patients.<br>• Young age, less severe illness, and trauma as the cause of admission were significantly prevalent in ARC patients**.<br>• ARC patients experienced lower serum VCM concentrations than the non-ARC group on the first, second, and third day of VCM therapy ($p < 0.01$)<br>• ARC patients reached therapeutic levels on day 3. There was a negative correlation between VCM concentrations and CrCl (r^2 −0.57)*

[a]ARC cut-off is reported in mL/min/1.73 m^2 unless indicated otherwise, * indicates $p < 0.001$ and ** indicates $p < 0.05$

Abbreviations: APACHE II score, acute physiology and chronic health evaluation II score; ARC, augmented renal clearance; ARCTIC, augmented renal clearance in trauma intensive care; aSAH, aneurysmal subarachnoid hemorrhage; AUC, area under the plasma concentration-time curve; BMI, body mass index; C/D, trough plasma concentration / maintenance dose ratio; CKD-EPI, Chronic Kidney Disease Epidemiology Collaboration; CL, clearance; CrCl, creatinine clearance; CRRT, continuous renal replacement therapy; C_{trough}, trough concentration; GC, Cockcroft-Gault; GFR, glomerular filtration rate; ICH, intracerebral hemorrhage; ICU, intensive care unit; IQR, interquartile range; IRF, impaired renal function; IV, intravenous; JPKD, JavaPK for Desktop; K_e, elimination rate constant; KeGFR, KineticGFR equation; LD, loading dose; MD, maintenance dose; MIC, minimum inhibitory concentration; NR, not reported; NRF, normal renal function; P, prospective; PD, pharmacodynamics; PK, pharmacokinetic; PopPK, population pharmacokinetic; q, dose frequency; R, retrospective; SCr, serum creatinine; SD, standard variation; TDM, therapeutic drug monitoring; $t_{1/2}$, half-life; TBI, traumatic brain injury; V_d, volume of distribution; VCM, vancomycin; VSCss, steady state trough vancomycin serum concentrations;

References

1. Ishigo, T.; Fujii, S.; Ibe, Y.; Aigami, T.; Nakano, K.; Fukudo, M.; Yoshida, H.; Tanaka, H.; Ebihara, F.; Maruyama, T.; et al. Flowchart for Predicting Achieving the Target Area under the Concentration-Time Curve of Vancomycin in Critically Ill Japanese Patients: A Multicenter Retrospective Study. *Journal of Infection and Chemotherapy* **2023**, S1341321X23002738, doi:10.1016/j.jiac.2023.11.001.
2. Ishigo, T.; Ibe, Y.; Fujii, S.; Kazuma, S.; Aigami, T.; Kashiwagi, Y.; Takada, R.; Takahashi, S.; Fukudo, M.; Toda, T. Effect of Renal Clearance on Vancomycin Area under the Concentration-Time Curve Deviations in Critically Ill Patients. *J Infect Chemother* **2023**, *29*, 769–777, doi:10.1016/j.jiac.2023.04.018.
3. Mikami, R.; Imai, S.; Hayakawa, M.; Sugawara, M.; Takekuma, Y. Clinical Applicability of Urinary Creatinine Clearance for Determining the Initial Dose of Vancomycin in Critically Ill Patients. *J Infect Chemother* **2022**, *28*, 199–205, doi:10.1016/j.jiac.2021.10.008.
4. Sahraei, Z.; Saffaei, A.; Alavi Darazam, I.; Salamzadeh, J.; Shabani, M.; Shokouhi, S.; Sarvmeili, N.; Hajiesmaeili, M.; Zangi, M. Evaluation of Vancomycin Pharmacokinetics in Patients with Augmented Renal Clearances: A Randomized Clinical Trial. *Front Pharmacol* **2022**, *13*, 1041152, doi:10.3389/fphar.2022.1041152.
5. Yu, Y.-X.; Lu, J.; Lu, H.; Li, L.; Li, J.-J.; Shi, L.; Duan, L.-F.; Zhuang, Z.-W.; Xue, S.-D.; Shen, Y.; et al. Predictive Performance of Reported Vancomycin Population Pharmacokinetic Model in Patients with Different Renal Function Status, Especially Those with Augmented Renal Clearance. *Eur. j. hosp. pharm., Sci. pract.* **2022**, *29*, e6–e14, doi:10.1136/ejhpharm-2020-002477.
6. Zhao, J.; Fan, Y.; Yang, M.; Liang, X.; Wu, J.; Chen, Y.; Guo, B.; Zhang, H.; Wang, R.; Zhang, F.; et al. Association between Augmented Renal Clearance and Inadequate Vancomycin Pharmacokinetic/Pharmacodynamic Targets in Chinese Adult Patients: A Prospective Observational Study. *Antibiotics (Basel)* **2022**, *11*, doi:10.3390/antibiotics11070837.
7. Zhao, S.; He, N.; Zhang, Y.; Wang, C.; Zhai, S.; Zhang, C. Population Pharmacokinetic Modeling and Dose Optimization of Vancomycin in Chinese Patients with Augmented Renal Clearance. *Antibiotics (Basel)* **2021**, *10*, doi:10.3390/antibiotics10101238.
8. Chen, Y.; Liu, L.; Zhu, M. Effect of Augmented Renal Clearance on the Therapeutic Drug Monitoring of Vancomycin in Patients after Neurosurgery. *J Int Med Res* **2020**, *48*, 300060520949076, doi:10.1177/0300060520949076.
9. Chu, Y.; Luo, Y.; Jiang, M.; Zhou, B. Application of Vancomycin in Patients with Augmented Renal Clearance. *Eur. j. hosp. pharm., Sci. pract.* **2020**, *27*, 276–279, doi:10.1136/ejhpharm-2018-001781.
10. Chu, Y.; Luo, Y.; Ji, S.; Jiang, M.; Zhou, B. Population Pharmacokinetics of Vancomycin in Chinese Patients with Augmented Renal Clearance. *J Infect Public Health* **2020**, *13*, 68–74, doi:10.1016/j.jiph.2019.06.016.
11. Helset, E.; Nordoy, I.; Sporsem, H.; Bakke, V.D.; Bugge, J.F.; Gammelsrud, K.W.; Zucknick, M.; von der Lippe, E. Factors Increasing the Risk of Inappropriate Vancomycin Therapy in ICU Patients: A Prospective Observational Study. *Acta Anaesthesiol Scand* **2020**, *64*, 1295–1304, doi:10.1111/aas.13658.
12. He, J.; Yang, Z.-T.; Qian, X.; Zhao, B.; Mao, E.-Q.; Chen, E.-Z.; Bian, X.-L. A Higher Dose of Vancomycin Is Needed in Critically Ill Patients with Augmented Renal Clearance. *Transl. androl. urol.* **2020**, *9*, 2166–2171, doi:10.21037/tau-20-1048.
13. Morbitzer K.; Jordan D.; Sullivan K.; Durr E.; Olm-Shipman C.; Rhoney D. Enhanced Renal Clearance and Impact on Vancomycin Trough Concentration in Patients with Hemorrhagic Stroke. *Pharmacotherapy* **2016**, *36*, e218, doi:10.1002/phar.1877.
14. Nelson, N.R.; Morbitzer, K.A.; Jordan, J.D.; Rhoney, D.H. The Impact of Capping Creatinine Clearance on Achieving Therapeutic Vancomycin Concentrations in Neurocritically Ill Patients with Traumatic Brain Injury. *Neurocrit Care* **2019**, *30*, 126–131, doi:10.1007/s12028-018-0583-z.
15. Ishii, H.; Hirai, K.; Sugiyama, K.; Nakatani, E.; Kimura, M.; Itoh, K. Validation of a Nomogram for Achieving Target Trough Concentration of Vancomycin: Accuracy in Patients With Augmented Renal Function. *Ther Drug Monit* **2018**, *40*, 693–698, doi:10.1097/FTD.0000000000000562.
16. Chu, Y.; Luo, Y.; Qu, L.; Zhao, C.; Jiang, M. Application of Vancomycin in Patients with Varying Renal Function, Especially Those with Augmented Renal Clearance. *Pharm biol* **2016**, *54*, 2802–2806.
17. Hirai, K.; Ishii, H.; Shimoshikiryo, T.; Shimomura, T.; Tsuji, D.; Inoue, K.; Kadoiri, T.; Itoh, K. Augmented Renal Clearance in Patients With Febrile Neutropenia Is Associated With Increased Risk for Subtherapeutic Concentrations of Vancomycin. *Ther Drug Monit* **2016**, *38*, 706–710.
18. Baptista, J.P.; Roberts, J.A.; Sousa, E.; Freitas, R.; Deveza, N.; Pimentel, J. Decreasing the Time to Achieve Therapeutic Vancomycin Concentrations in Critically Ill Patients: Developing and Testing of a Dosing Nomogram. *Crit Care* **2014**, *18*, 654, doi:10.1186/s13054-014-0654-2.
19. Campassi, M.L.; Gonzalez, M.C.; Masevicius, F.D.; Vazquez, A.R.; Moseinco, M.; Navarro, N.C.; Previgliano, L.; Rubatto, N.P.; Benites, M.H.; Estenssoro, E.; et al. [Augmented Renal Clearance in Critically Ill Patients: Incidence, Associated Factors and Effects on Vancomycin Treatment]. *Rev. bras. ter. intensiva* **2014**, *26*, 13–20.
20. Minkute, R.; Briedis, V.; Steponaviciute, R.; Vitkauskiene, A.; Maciulaitis, R. Augmented Renal Clearance--an Evolving Risk Factor to Consider during the Treatment with Vancomycin. *J Clin Pharm Ther* **2013**, *38*, 462–467, doi:10.1111/jcpt.12088.
21. Baptista, J.P.; Sousa, E.; Martins, P.J.; Pimentel, J.M. Augmented Renal Clearance in Septic Patients and Implications for Vancomycin Optimisation. *Int J Antimicrob Agents* **2012**, *39*, 420–423, doi:10.1016/j.ijantimicag.2011.12.011.
